# Vaccine Virus

**Published:** 1873-05

**Authors:** 


### Editorial.

### *Vaccine Virus.*

In common with our brethren of the profession, we have experienced great difficulty in procuring reliable vaccine virus, even for our own use in private practice. It is, therefore, with great pleasure that we now call attention to a circular in our advertising pages, announcing a new source of supply.

From the testimonials and letters which have been presented to us, we believe that our friends may rely upon our advertisers as performing exactly what they promise, and furnishing precisely what is asked for. It is a good time now to test whether the virus, fresh from the heifer, is a better prophylactic than that which has for generations, perhaps, been passing through the human body. Very many bits of scab will make a *sizable* sore when tucked under the epidermis, but by no means prevent small-pox. To accommodate our subscribers and others corresponding with, or visiting, our publishers at their mammoth bookstore, they have consented to act as agents, and supply calls for the guaranteed virus. Address, as upon the cover: W. B. KEEN, COOKE & Co., 113 and 115 State Street, Chicago.

